# Stress Granules Modulate SYK to Cause Tau-Associated Neurocognitive Deterioration in 5XFAD Mouse After Anesthesia and Surgery

**DOI:** 10.3389/fnagi.2021.718701

**Published:** 2021-08-27

**Authors:** Yang Shen, Tong Zhang, Yinglin Zhang, Yinuo Wang, Junyan Yao

**Affiliations:** Department of Anesthesiology, Shanghai General Hospital, Shanghai Jiao Tong University School of Medicine, Shanghai, China

**Keywords:** Alzheimer’s disease, postoperative cognitive dysfunction, stress granules, SYK tyrosine kinase, tauopathy, 5XFAD, cognitive decline

## Abstract

**Background:**

Alzheimer’s disease (AD) is the most common type of dementia. However, no curative therapy has been found effective to slow down the process of AD. It is reported that anesthesia and surgery will induce neurocognitive deterioration in AD, but the mechanism is not quite clear. In this study, we aim to compare the cognitive impairment between 5XFAD transgenic (Tg) mice and its littermate (LM) after isoflurane anesthesia and surgery to clarify the specific impacts of anesthesia and surgery on individuals with AD and to explore the mechanisms.

**Methods:**

We performed abdominal surgery in cognitively impaired, 4-month-old female 5XFAD mice and LM control mice. Isoflurane anesthesia (1.4%) was induced and maintained over 2 h. Open field and fear conditioning tests were conducted on 1, 3 and 7 days after anesthesia and surgery. The total distance, velocity and freezing time were the major outcomes. P-tau (AT8), tau oligomers (T22), stress granules (SGs), the SYK tyrosine kinase and p-SYK in the hippocampus at postoperative day 1 were evaluated by Western Blot assays. The colocalization of SGs, SYK, p-SYK, and neurons in the hippocampus section was assessed using qualitative immunofluorescence.

**Results:**

In the open field test, no difference between the distance moved and the velocity of LM mice and 5XFAD Tg mice were found on day 1 after anesthesia and surgery. 5XFAD Tg mice exhibited reduced freezing time of fear conditioning context test on postoperative day 3, but not on day 7; the LM mice showed no changes in FCTs. Furthermore, p-tau, tau oligomers, SGs, SYK and p-SYK were evident in the hippocampus region of 5XFAD Tg mice on a postoperative day 1. In addition, SGs, SYK, p-SYK were colocalized with hippocampus neurons, as shown by immunofluorescence.

**Conclusion:**

This study demonstrates that anesthesia and surgery may induce tau-associated neurocognitive deterioration in individuals with AD. The mechanism under it may be associated with SGs and the tyrosine kinase, SYK. After anesthesia and surgery, in 5XFAD Tg mice, SGs were formed and SYK was phosphorylated, which may contribute to the phosphorylation of tau protein. This study provided hints that individuals with AD may be more vulnerable to anesthesia and surgery.

## Introduction

Alzheimer’s disease (AD) is the most common cause of dementia, accounting for an estimated 60–80% (Alzheimer’s Association, 2020). Worldwide, about 50 million people were living with AD in 2019, and this number is projected to triple by 2050 (Alzheimer’s Disease International, 2019). However, AD has long been considered to be neither preventable nor treatable (Livingston et al., 2017). As estimated by World Health Organization, there have been about 312.9 million surgeries in 2012 globally (Weiser et al., 2016). As overall life expectancy and the advances in surgical techniques have increased, more and more elderly patients are undergoing anesthesia and surgery. Besides, elderly surgical patients are more likely to have preexisting AD or be at risk for developing it (Işik, 2015). On the other hand, there are clinical studies that fail to support the association of anesthesia and surgery with AD (Avidan and Evers, 2011; Sprung et al., 2013, 2020; Aiello Bowles et al., 2016; Silber et al., 2020). However, some animal experiments have been reported where individuals with AD may develop cognitive impairment after anesthesia and surgery, but the mechanism is not clear (Zhang et al., 2017; Miao et al., 2018; Kim et al., 2021).

Recent advances indicate that the response of RNA metabolism to stress has an important role in the pathophysiology of neurodegenerative disease, particularly AD (Wolozin and Ivanov, 2019). Stress granules (SGs) are transient membraneless organelles that control the utilization of mRNA during stress (Wolozin and Ivanov, 2019). However, evidence suggests that chronic stresses associated with aging, such as AD, lead to chronic and persistent SGs that act as a nidus for the aggregation of disease-related proteins (Wolozin and Ivanov, 2019). Anesthesia/surgery is an acute stress to the body and whether this kind of acute stress would deteriorate the pathology of AD is unknown.

It has been reported that hyperphosphorylation of SYK can result in the aggregation and phosphorylation of tau protein through the SYK/PKA/GSK3β pathway (Paris et al., 2014; Schweig et al., 2017). Furthermore, evidence suggests that SYK can be recruited and activated by SGs, enhance the formation of SGs, and stimulate the production of reactive oxygen and nitrogen species in microglia of AD (Ghosh and Geahlen, 2015). Taken together, we employed the 5XFAD mice in the studies to illustrate the potential role of tau, SGs and SYK in the anesthesia/surgery neurotoxicity in AD.

## Materials and Methods

### Animals and Disease Model

The animal protocol was approved (Protocol number: 2019AW027, Shanghai, China) by the Shanghai General Hospital Clinical Center Laboratory Animal Welfare and Ethics Committee. Efforts were made to minimize the number of animals used. The AD transgenic (Tg) mice, 5XFAD, were purchased from Jackson Lab [B6SJL Tg (APPSwF1Lon, PSEN1^∗^M146L^∗^L286V) 6799Vas/Mmjax; Stock Number: 006554] and maintained in Shanghai Donghua University SPF Animal Center until 4 months of age. The mice were housed in a controlled environment (20–22°C; 12 h of light/dark on a reversed light cycle) for 7 days prior to the studies. In this study, littermate (LM) mice were used as the littermate control mice of 5XFAD Tg mice. C57BL/6J female mice and 5XFAD Tg male mice mated to obtain progeny mice. When the progeny mice were 1 month old, their tails were sampled for genotyping. The genotyping of progeny mice was performed by PCR analysis of tail sample DNA, according to the protocol of the supplier. The progeny mice with the expression of both APP (377 bp) and PS1 (608 bp) were identified as 5XFAD Tg mice, whereas ones with the expression of only reference DNA (325 bp) were employed as LM mice in this study. The mice were randomly assigned to either the anesthesia/surgery group or the control condition group. The anesthesia/surgery was performed between 9 a.m. and 12 p.m. A simple laparotomy was made under 1.4% isoflurane anesthesia (anesthesia/surgery). Specifically, anesthesia was induced and maintained with 1.4% isoflurane in 100% oxygen in a transparent acrylic chamber. 15 min after the induction, each of the mice was moved out of the chamber, and isoflurane anesthesia was maintained *via* a cone device. To monitor the concentration of isoflurane, we inserted a 16-gage needle into the cone near the nose of the mouse. A longitudinal midline incision was made from the xiphoid to the 0.5 cm proximal pubic symphysis on the skin, abdominal muscles and peritoneum. Then, the incision was sutured layer by layer with 5–0 Vicryl thread. The procedure for each mouse lasted about 10 min, and the mouse was put back into the anesthesia chamber for up to 2 h to receive the rest of the anesthesia consisting of 1.4% isoflurane in 100% oxygen. We used this combination of anesthesia and surgery treatment in the studies because it has been reported that the anesthetic could induce cognitive impairment and surgery might potentiate the anesthesia-induced neurotoxicity and neurobehavioral deficits (Zhang et al., 2017; Miao et al., 2018). The temperature of the anesthetizing chamber was controlled to maintain the rectal temperature of the mice at 37 ± 0.5°C during the anesthesia/surgery procedure. After recovering from the anesthesia, each mouse was returned to a home cage with food and water available *ad libitum*. The mice in the control group (food and water available *ad libitum*) were placed in their home cages with 100% oxygen for 2 h.

### Behavioral Analysis

**FIGURE 1:**
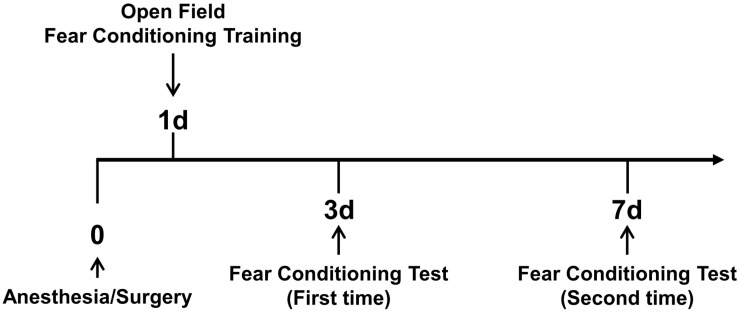
Overview of behavioral analysis timeline. On the first day after anesthesia and surgery, the open field test and fear conditioning training were conducted. Then, on days 3 and 7 after anesthesia and surgery, fear conditioning tests were conducted.

In this study, we used two behavioral analyses to assess the cognitive changes of 5XFAD Tg mice and LM mice after anesthesia/surgery. The procedure of behavioral analysis is shown in Figure 1.

#### Open Field Test

To test the effects of anesthesia/surgery on locomotor activity, the open-field test was used. The test apparatus was a square arena (40 cm × 40 cm × 60 cm). The mice were gently placed in the center of the field, and behavioral paraments were recorded for 300 s using the EthoVision video tracking system (version 3.0, Noldus, Wageningen, Netherlands). The distance moved (cm/5 min) and velocity (cm/s) were recorded.

#### Fear Conditioning Test

The fear conditioning test (FCT) training was performed on day 1 after the anesthesia/surgery. Each mouse was allowed to explore the FCT chamber for 180 s before the presentation of a pulsating tone (80 db, 3.6 kHz) that persisted for 60 s. The tone was followed immediately by a mild foot shock (0.8 mA for 0.5 s). The stimulation described above was repeated one more time with an interval of 120 s. The context test was performed on day 3 and then day 7 after the anesthesia/surgery, respectively. Each mouse was allowed to stay in the chamber for a total of 390 s. The function of learning and memory in the context test was assessed by measuring the amount of time the mouse demonstrated “freezing behavior,” which is defined as a completely immobile posture except for respiratory efforts during the second 180 s. The tone test was also performed on days 3 and 7 after the anesthesia/surgery. Each mouse was allowed to stay in the chamber for a total of 390 s. The same tone was presented for the second 180 s without the foot shock. The function of learning and memory in the tone test was assessed by measuring the amount of time the mouse demonstrated “freezing behavior,” defined as a completely immobile posture except for respiratory efforts during the second 180 s. The “freezing behavior” was analyzed by Video Freeze (freezing on the threshold: 18 pixels/f; minimum freezing duration: 30 f). The freezing time was presented in seconds.

### Western Blot Analysis

Hippocampus protein was harvested and homogenized in RIPA lysis buffer (NCM Biotech, Suzhou, China) containing protease (NCM Biotech, Suzhou, China) and phosphatase inhibitor (NCM Biotech, Suzhou, China). The protein concentration was determined with a bicinchoninic acid assay kit (Thermo Fisher Scientific, MA, United States). Western blotting was performed as described previously. Briefly, 10 μg of protein was loaded per lane on a 10% SDS-PAGE. The membrane blots were saturated with blocking buffer (NCM Biotech, Suzhou, China) for 15 min at room temperature and then incubated overnight at 4°C with antibodies against GAPDH (1:1000, Proteintech, Wuhan, China), T22 (1:1000, Millipore, MA, United States), AT8 (1:1000, Thermo Fisher Scientific, MA, United States), G3BP (1:1000, Abcam, Cambridge, United Kingdom), SYK (1:1000, Abcam, Cambridge, United Kingdom), and p-SYK (1:1000, Abcepta, Suzhou, China). The gray intensity of proteins was measured using Image J software (United States National Institutes of Health).

### Immunofluorescence

The mice were anesthetized with 1% pentobarbital sodium and perfused with saline for a few minutes. Then, the mice were perfused with 4% paraformaldehyde (PFA) in phosphate-buffered saline (PBS). Brains were dissected and soaked overnight at 4°C in 4% PFA, and then soaked in 30% sucrose at 4°C for 48 h. Brain slices (25 μm) were harvested and incubated with primary and secondary antibodies, and then washed in PBST and flat-mounted. The primary antibodies included anti-NeuN (Millipore, MA, United States), anti-G3BP (Abcam, Cambridge, United Kingdom), anti-SYK (Abcam, Cambridge, United Kingdom), and anti-p-SYK (Abcepta, Suzhou, China). The secondary antibodies included donkey anti-rabbit IgG H&L (Alexa Fluor 488, Jackson Immuno Research, PA, United States), donkey anti-rabbit IgG H&L (Alexa Fluor 594, Thermo Fisher Scientific, MA, United States), and donkey anti-guinea pig (Alexa Fluor 647, Abcam, Cambridge, United Kingdom) antibodies. Primary antibodies were diluted at 1:100 for usage, and the secondary antibodies were applied at a dilution of 1:200. In each group, three hippocampus flat mounts were performed and observed under a confocal microscope (LEICA TCS SP8 X, Germany).

### Statistical Analysis

Representative results are shown in the figures. The sample size is chosen as generally required for basic research. For behavioral studies, the sample size was 12 per group. For Western blotting, at least five hippocampi per animal group were used. Samples are randomly assigned to experimental groups. Data are presented as mean ± SEM and were analyzed statistically using one-way ANOVA, two-way ANOVA, or two-tailed Student’s *t*-test. *P*-values < 0.05 were considered statistically significant.

## Results

### Anesthesia/Surgery Did Not Impact the Locomotor Activity of LM Mice and 5XFAD Tg Mice

**FIGURE 2:**
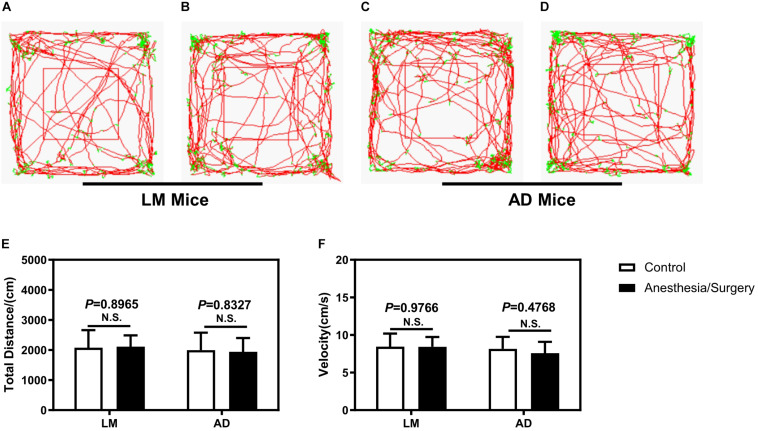
The effect of anesthesia and surgery on the locomotor activity of LM mice and 5XFAD Tg mice. The movement trajectory **(A–D)**, total distance **(E)**, and velocity **(F)** of LM mice and 5XFAD Tg mice in the control and anesthesia/surgery groups. The movement trajectory of LM mice and 5XFAD Tg mice were both evenly distributed, and no difference was found in the total distance and velocity of LM mice and 5XFAD Tg mice between the control and anesthesia/surgery groups. Results are expressed as mean ± SEM, *n* = 12. Student *t*-test. N.S., *P* > 0.05 vs. untreated controls.

To eliminate the impacts of locomotor activity on the assessment of cognitive function between 5XFAD Tg mice and LM mice, first, we carried out an open-field test. As shown in Figure 2, the movement trajectory of LM mice and 5XFAD Tg mice were both evenly distributed. In addition, we found no difference between the distance moved and velocity of 4-month female 5XFAD Tg mice and LM mice regardless of anesthesia/surgery (Figures 2E,F).

### Anesthesia/Surgery-Induced Cognitive Impairment in Female 5XFAD Tg Mice

**FIGURE 3:**
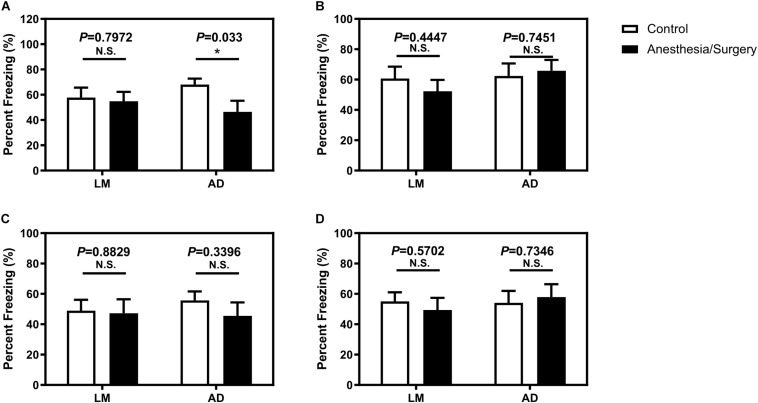
The effect of anesthesia and surgery on the cognition of LM mice and 5XFAD Tg mice. The percent freezing of LM mice and 5XFAD Tg mice in control and anesthesia/surgery group on day 3 after anesthesia and surgery in context **(A)** and tone **(B)** FCT. The percent freezing of LM mice and 5XFAD Tg mice in control and anesthesia/surgery group on day 7 after anesthesia and surgery in context **(C)** and tone **(D)** FCT. The FCT revealed that LM mice showed no cognitive dysfunction both on day 3 and day 7 after anesthesia and surgery, but 5XFAD Tg mice showed a cognitive decline in context, but not tone test, on day 3 after anesthesia and surgery. 5XFAD Tg mice showed no cognitive impairment in both context and tone test on day 7 after anesthesia and surgery. Results are expressed as mean ± SEM, *n* = 12. Student *t*-test. *P < 0.05 vs. untreated controls. N.S., *P* > 0.05 vs. untreated controls.

Given no changes of locomotor activity between female LM mice and 5XFAD Tg mice, we carried out FCT to detect the acute cognitive function after anesthesia/surgery. We found that the anesthesia/surgery significantly decreased the freezing time in the context test, instead of the tone test of FCT as compared with the control group in the 4-month female 5XFAD Tg mice (Figures 3A,B) 3 days after the anesthesia/surgery. However, 7 days after the anesthesia/surgery, the anesthesia/surgery did not significantly change the freezing time in both context and tone test as compared with the control group in 4-month female 5XFAD Tg mice (Figures 3C,D). These data suggest that the anesthesia/surgery could induce hippocampus-dependent cognitive impairment 3 days after, but not 7 days after the anesthesia/surgery in the 4-month female 5XFAD Tg mice.

In the age-matched female LM mice, however, we found that the anesthesia/surgery did not significantly change the freezing time of both context and tone test of FCT 3 or 7 days after the anesthesia/surgery as compared with the control group (Figure 3). These data suggested that the anesthesia/surgery did not induce cognitive impairment as compared with the control condition in the age-matched female LM mice.

### Anesthesia/Surgery Increased the Levels of p-Tau and Tau Oligomers in the Hippocampus of Female 5XFAD Tg Mice

**FIGURE 4:**
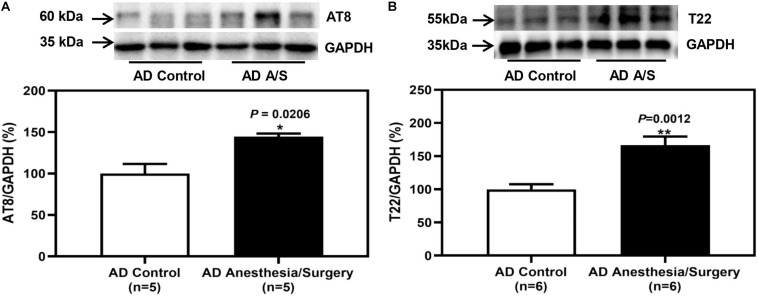
The effect of anesthesia and surgery on the levels of p-tau and tau oligomers in the hippocampus of 5XFAD Tg mice. **(A,B)** The difference in brain amounts of phosphorylated tau at Ser202 and Thr205 sites (AT8) and tau oligomers (T22) in the hippocampus of postoperative 24 h of 5XFAD Tg mice in control and anesthesia/surgery group. In 5XFAD Tg mice, the levels of AT8 and T22 in the hippocampus were significantly increased at postoperative 24 h. Results are expressed as mean ± SEM. Student *t*-test. **P < 0.005 vs. untreated controls. *P < 0.05 vs. untreated controls.

As the anesthesia/surgery-induced acute cognitive dysfunction in 4-month female 5XFAD Tg mice, next, we determined the potential cellular mechanisms. To evaluate whether the impairment of neurocognitive function is related to changes in hippocampus amounts of p-tau and tau oligomer in 5XFAD Tg mice, we first tested the amounts of these proteins in 5XFAD Tg mice after anesthesia and surgery. Western blot analysis of protein expression revealed that after anesthesia and surgery, p-tau (Figure 4A) and tau oligomers (Figure 4B) in the hippocampus of 5XFAD Tg mice were significantly increased.

### Anesthesia/Surgery-Induced the Formation of SGs in Neurons of the Hippocampus of Female 5XFAD Tg Mice

**FIGURE 5:**
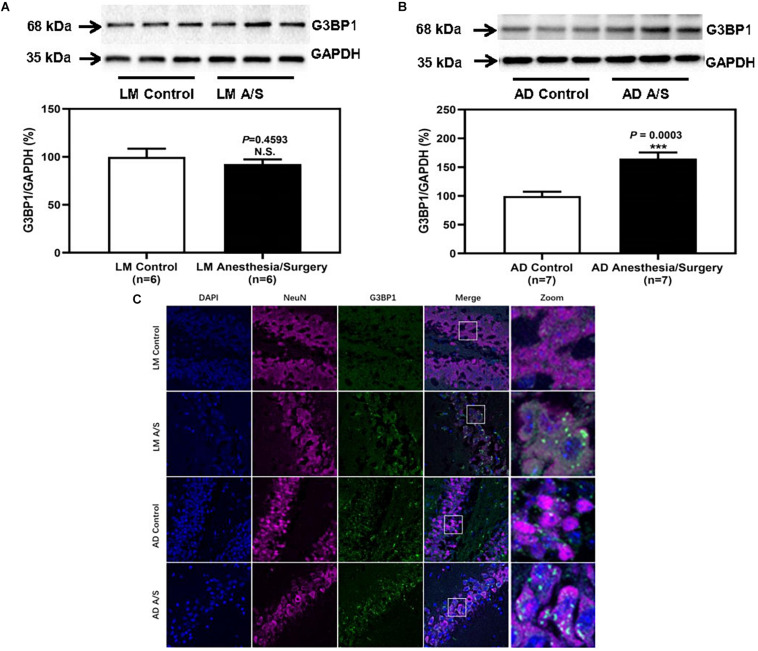
The effect of anesthesia and surgery on the levels of SGs in the hippocampus of LM mice and 5XFAD Tg mice. G3BP1 expression in the hippocampus of postoperative 24 h of LM mice **(A)** and 5XFAD Tg mice **(B)** in control and anesthesia/surgery group. **(C)** Immunostaining of G3BP1 positive neurons in the hippocampus of LM mice and 5XFAD Tg mice in control and anesthesia/surgery group. The hippocampus level of G3BP1 was significantly increased in 5XFAD Tg mice but not LM mice at postoperative 24 h. The immunostaining of G3BP1 showed that G3BP1 and hippocampus neuron (NeuN) were colocalized. Results are expressed as mean ± SEM. Student’s *t*-test. N.S., *P* > 0.05 vs. untreated controls. ***P < 0.0005 vs. untreated controls.

As we indicated above, SGs are cytoplasmic members of the RNA granule family and have been implicated in the pathogenesis of AD. Therefore, we determined the effects of the anesthesia/surgery on the amount of SGs in the hippocampus of LM mice and 5XFAD Tg mice. The immunoblotting of G3BP1, a marker of SGs, showed that the anesthesia/surgery increased the density of the bands representing G3BP1 in the hippocampus of 4-month female 5XFAD Tg mice (Figure 5B). However, for LM mice, compared with LM control mice, the expression of G3BP1 of LM mice treated by anesthesia and surgery did not increase (Figure 5A). The immunostaining of G3BP1 showed that G3BP1 and hippocampus neuron (NeuN) were colocalized (Figure 5C).

### SYK Was Activated and Colocalized With SGs in Neurons of the Hippocampus of Female 5XFAD Tg Mice

**FIGURE 6:**
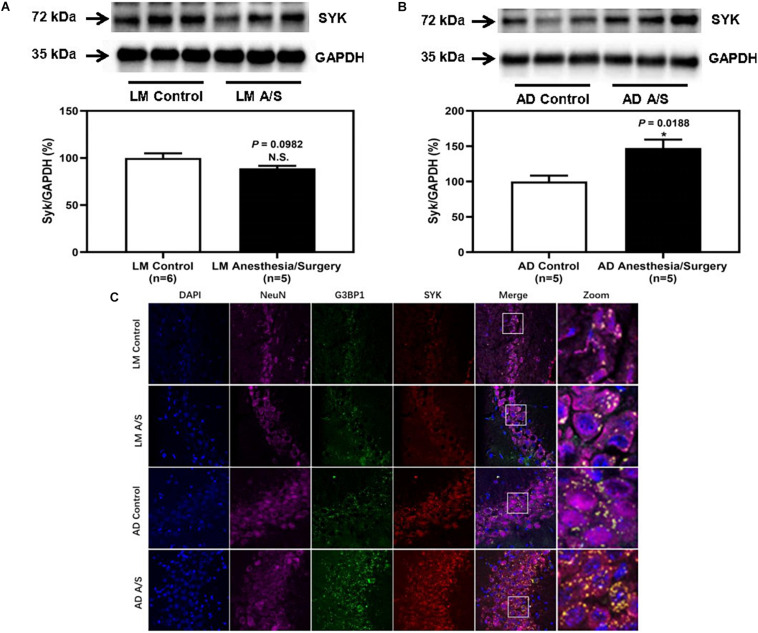
The effect of anesthesia and surgery on the levels of SYK in the hippocampus of LM mice and 5XFAD Tg mice. SYK expression in the hippocampus of postoperative 24 h of LM mice **(A)** and 5XFAD Tg **(B)** mice in control and anesthesia/surgery group. **(C)** Immunostaining of SYK and G3BP1 positive neurons in the hippocampus of LM mice and 5XFAD Tg mice in control and anesthesia/surgery group. The hippocampus level of SYK was significantly increased in 5XFAD Tg mice but not LM mice at postoperative 24 h. The immunostaining of SYK and G3BP1 showed that SYK, G3BP1, and hippocampus neurons (NeuN) were colocalized. Results are expressed as mean ± SEM. Student *t*-test. N.S., *P* > 0.05 vs. untreated controls. *P < 0.05 vs. untreated controls.

**FIGURE 7:**
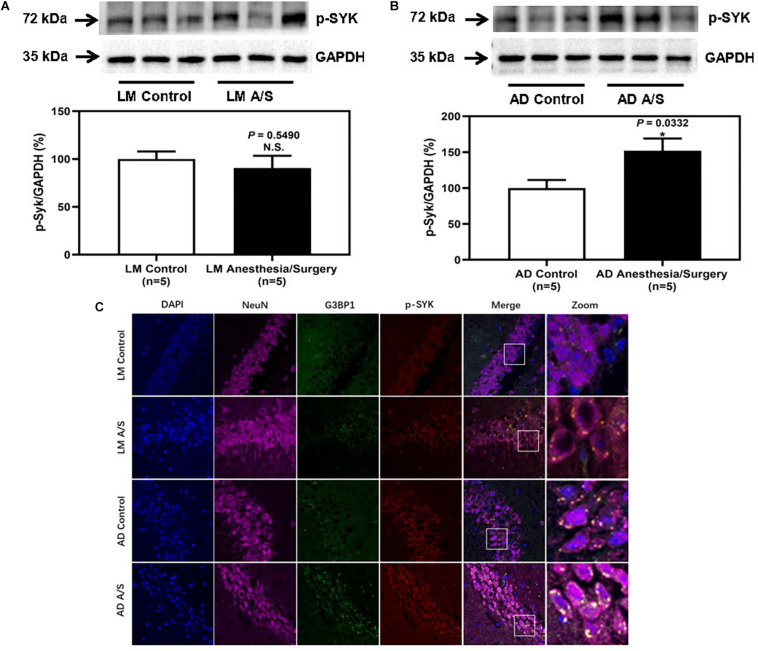
The effect of anesthesia and surgery on the levels of p-SYK in the hippocampus of LM mice and 5XFAD Tg mice. P-SYK expression in the hippocampus of postoperative 24 h of LM mice **(A)** and 5XFAD Tg mice **(B)** in control and anesthesia/surgery group. **(C)** Immunostaining of p-SYK and G3BP1 positive neurons in the hippocampus of LM mice and 5XFAD Tg mice in control and anesthesia/surgery group. The hippocampus level of p-SYK was significantly increased in 5XFAD Tg mice but not LM mice at postoperative 24 h. The immunostaining of p-SYK and G3BP1 showed that p-SYK, G3BP1, and hippocampus neurons (NeuN) were colocalized. Results are expressed as mean ± SEM. Student *t*-test. N.S., *P* > 0.05 vs. untreated controls. *P < 0.05 vs. untreated controls.

To confirm that the protein level of SYK was increased in 5XFAD Tg mice after anesthesia and surgery, we used the immunoblotting method to determine the protein level of SYK. The anesthesia/surgery did not significantly increase the protein level of SYK in LM mice as compared with the control group (Figure 6A). However, the immunoblotting of SYK showed that the anesthesia/surgery significantly increased the density of the bands representing SYK in the hippocampus of 4-month female 5XFAD Tg mice as compared with the control group (Figure 6B). Then, we used the immunofluorescence method to explore whether SYK can also be recruited to SGs. As shown in Figure 6C, SYK was colocalized with G3BP1 in SGs in both LM and 5XFAD Tg mice regardless of anesthesia/surgery. We then ask if SYK colocalized to SGs was activated. Since phosphorylated SYK (p-SYK) is the form of activated SYK, we detected the p-SYK. Firstly, the immunoblotting of p-SYK showed that the phosphorylation of SYK was increased in 5XFAD Tg mice treated with anesthesia/surgery as compared with the control 5XFAD Tg mice (Figure 7B), whereas anesthesia and surgery had no effect on the phosphorylation of SYK in LM mice (Figure 7A). We used immunofluorescence to determine the colocalization of p-SYK and SGs. The results showed that SYK was activated and colocalized with SGs in neurons of the hippocampus in 5XFAD Tg mice after anesthesia and surgery (Figure 7C).

## Discussion

The findings that the anesthesia/surgery did not significantly affect the total distance and velocity of the open-field test indicated that the anesthesia/surgery did not impair the locomotor activity (Figure 2). Therefore, the observed shorter freezing time of the fear conditioning context test was not due to the changes in locomotor activity. The behavior tests of fear conditioning suggest that isoflurane plus surgery caused a reduced freezing time of fear conditioning context test in 5XFAD Tg mice but not in LM mice (Figure 3). These results were consistent with previous studies (Miao et al., 2018) and with Zhang et al. (2017) reported that after sevoflurane anesthesia and abdominal surgery, 5XFAD Tg female, but not LM mice, were of cognitive dysfunction in fear conditioning context test. In another study, Miao et al. (2018) found that isoflurane, but not desflurane plus abdominal surgery, could cause longer escape latency and distance of Barnes maze probe test, which suggested cognitive impairment in 5XFAD Tg mice. In this study, we used the same surgery type but different inhalation anesthetics and found that 5XFAD Tg mice, instead of LM mice, were of cognitive dysfunction. Therefore, inhalation anesthetics plus abdominal surgery could induce cognitive impairment in 5XFAD Tg mice. Furthermore, Miao et al. reported that isoflurane, instead of desflurane, was detrimental, whereas Zhang et al. reported that sevoflurane was harmful to the cognition of mice with AD, suggesting that different types of inhalation anesthetics may have different impacts on the cognition of AD mice. However, more researches, especially prospective controlled clinical trials are needed to confirm this review to screen which inhalation anesthetics are more suitable for patients with AD.

In the Barnes maze probe test, until 28 days after anesthesia and surgery, 5XFAD Tg mice showed a cognitive decline (Miao et al., 2018). Compared with the FCT, the Barnes maze is a behavioral test intended to test the spatial memory of rodents and has no stress to rodents. Since the FCT involves pain factors and is stressful for rodents, it can test memory in a short time (Wahl et al., 2017). In general, our results are consistent with other studies that anesthesia plus surgery may promote AD development by aggravating cognitive dysfunction.

Amyloid plaques and neurofibrillary tangles are characteristics of AD, which are composed of Aβ peptide and tau protein, respectively (Scheltens et al., 2016). In recent years, most of the new Aβ-targeting therapies have failed in clinical trials. Thus, therapies for AD in clinical trials are gradually shifting from Aβ-targeting therapies to tau-targeting immunotherapies (Long and Holtzman, 2019). In this study, we determined that after anesthesia and surgery, it was tau, but not Aβ that increased significantly. We first analyzed the Aβ oligomers level and the tau phosphorylation of 5XFAD Tg mice after anesthesia and surgery. We found that Aβ oligomers did not increase significantly (data not shown). On the contrary, in 5XFAD Tg mice, anesthesia and surgery-induced abnormal tau phosphorylation, as evidenced by immunoblotting of the hippocampus with antibody AT8 (Figure 4A). Then, we tested the toxic tau oligomers with antibody T22 and found that T22 was significantly increased (Figure 4B). Previous studies have suggested that hyperphosphorylated tau proteins will gradually deposit into tau oligomers, which produce neurotoxicity (Castellani and Perry, 2019). Therefore, it is indicated that anesthesia and surgery may affect the degree of tau hyperphosphorylation and lead to cognitive impairment.

Also, other studies have reported that tau hyperphosphorylation is associated with memory decline after inhalation anesthetics exposure. Tan et al. (2010) found that isoflurane anesthesia combined with hypothermia could increase tau phosphorylation at the Thr205 and Ser396 sites in the hippocampus of WT mice, while Dong et al. (2012) and Li et al. (2014) observed that increased p-tau at the Ser262 site is associated with memory impairment in WT and AD Tg mice (APP695) after isoflurane exposure, respectively. Recently, Dong et al. (2021) used a new approach called nanobeam-sensor to demonstrate that sevoflurane could lead to tau exiting from neurons upon phosphorylation, traveling through both extracellular vesicles (EVs) and non-EVs routes, and then entering microglia, leading to the generation of IL-6 and cognitive impairment *in vitro* and *in vivo* experiments. These data indicated that tau also played an important role in anesthesia-induced cognitive impairment.

Stress granules are membraneless organelles, which are composed of messenger ribonucleoproteins and RNA-binding proteins (RBPs). In response to biotic or abiotic stresses, SGs quickly assemble within minutes to hours, and quickly dissolve when the stress is removed. But chronic illness, such as AD, produces persistent stress that allows time for SGs to mature into more stable complexes. SGs may be an initial position where pathological tau proteins aggregate (Apicco et al., 2018; Silva et al., 2019; Wolozin and Ivanov, 2019). Therefore, when we found that the levels of p-tau and tau oligomers were significantly increased after anesthesia and surgery, we paid attention to the influence of anesthesia and surgery on SGs. Anesthesia/surgery was a kind of acute stress for the body, and what effects it would have on SGs was unknown. SGs can be defined by the presence of core nucleating RBPs, such as T cell intracellular antigen 1 (TIA1), G3BP1, etc. (Wolozin and Ivanov, 2019). Among these SGs markers, G3BP1 is an important assembly factor of SGs (Yang et al., 2020). Thus, in this study, we used G3BP1 as a marker of SGs and demonstrated that anesthesia and surgery-induced increased assembly of SGs in 5XFAD Tg mice but not LM mice. Also, immunofluorescence staining was performed to identify the SGs in neurons of the hippocampus, and results showed that G3BP1 was colocalized with neurons. These results suggested that SGs in hippocampus neurons significantly increased in 5XFAD mice after anesthesia/surgery. Then, we wondered whether anesthesia/surgery promotes the progression of AD through the regulation of SGs and how SGs regulate the pathological changes of tau protein. It has been reported that overactivation of SYK can exacerbate the hyperphosphorylation and aggregation of tau proteins (Paris et al., 2014). Activated SYK can inactive the function of protein kinase A (PKA) to enhance the downstream kinase, GSK3β, and GSK3β is one of the main kinases, phosphorylating tau proteins at Ser202 and Ser396/Ser404 sites (Paris et al., 2014; Schweig et al., 2017). Furthermore, Ghosh and Geahlen (2015) found that in microglia, SYK was recruited and activated in SGs. Hence, we tested the expression of SYK in the hippocampus region and found that anesthesia and surgery increased the expression and activation of SYK. Consistently, we showed that SYK was co-localized with SGs in neurons. In addition, recent studies reported that TIA1 could interact with tau protein to induce AD-related neurotoxicity (Apicco et al., 2018). Also, TIA1 is involved in neuronal response to spreading tau oligomers (Jiang et al., 2019). As we indicated above, tau oligomers (T22) were significantly increased in 5XFAD Tg mice after anesthesia and surgery. Thus, whether TIA1 is facilitated in the deterioration of tauopathy caused by anesthesia and surgery still needs more exploration.

In conclusion, we found that abdominal surgery under isoflurane (anesthesia/surgery) was able to induce hippocampus-dependent cognitive decline and an increase in hippocampus level of tau oligomers and p-tau in 4-month female 5XFAD Tg mice, but not LM mice. Meanwhile, we also found increased SGs, SYK and p-SYK in 5XFAD Tg mice after anesthesia and surgery for the first time, which may be related to the cognitive decline of 5XFAD mice with anesthesia and surgery. However, due to the experimental conditions, we have been unable to obtain enough Tg mice with AD to investigate the causal relationship between these phenomena. This is a limitation of this study. Although this study gives us hints that patients with AD may be more vulnerable to develop postoperative cognitive decline than non-demented patients, there is still a long way to go to verify it in human beings.

## Data Availability Statement

The original contributions presented in the study are included in the article/Supplementary Material, further inquiries can be directed to the corresponding author/s.

## Ethics Statement

The animal study was reviewed and approved by Shanghai General Hospital Clinical Center Laboratory Animal Welfare and Ethics Committee.

## Author Contributions

JY designed the study. YS, TZ, and YZ performed the experimental phase. YS and TZ collected and analyzed data. YS and JY drafted the manuscript. YW contributed with reagents, materials, and analysis tools. YS, TZ, YZ, YW, and JY have collaborated and approved the final manuscript version.

## Conflict of Interest

The authors declare that the research was conducted in the absence of any commercial or financial relationships that could be construed as a potential conflict of interest.

## Publisher’s Note

All claims expressed in this article are solely those of the authors and do not necessarily represent those of their affiliated organizations, or those of the publisher, the editors and the reviewers. Any product that may be evaluated in this article, or claim that may be made by its manufacturer, is not guaranteed or endorsed by the publisher.
