# Supplementary material for: Stress Granules Modulate SYK to Cause Tau-Associated Neurocognitive Deterioration in 5XFAD Mouse After Anesthesia and Surgery
Source: Front Aging Neurosci. 2021 Aug 27;13:718701. doi: 10.3389/fnagi.2021.718701 (PMC8430336; doi:10.3389/fnagi.2021.718701)

AT8 Show WB picture (blue arrow points out the standard markers)
The upper marker is 72kda, the other one is 55kda.

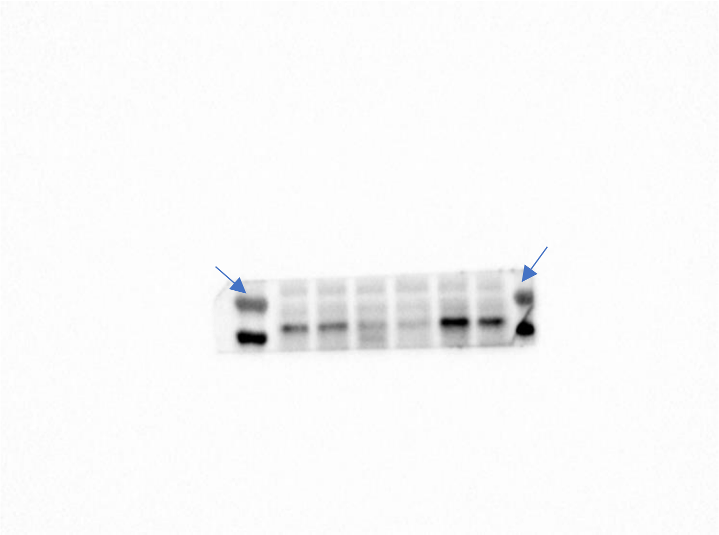

Its original internal reference GPADH (blue arrow points out the standard markers)
The upper marker is 43kda, the other one is 33kda.

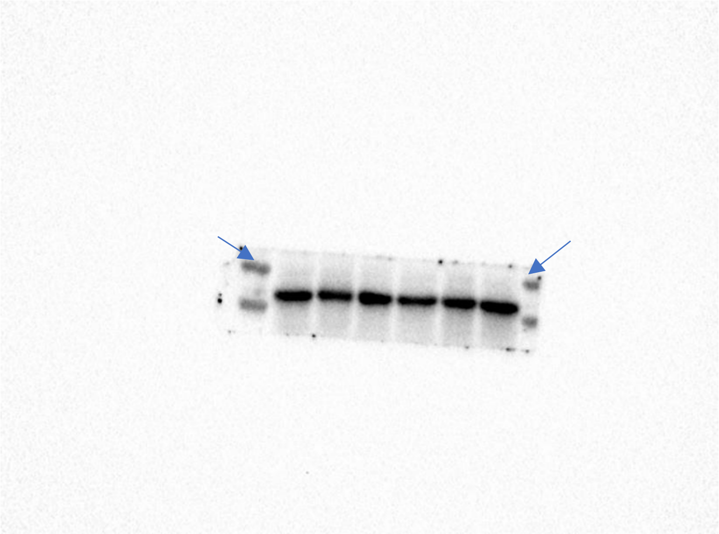

AT8 WB picture for statistical analysis (blue arrow points out the standard markers)
The upper marker is 72kda, the other one is 55kda.

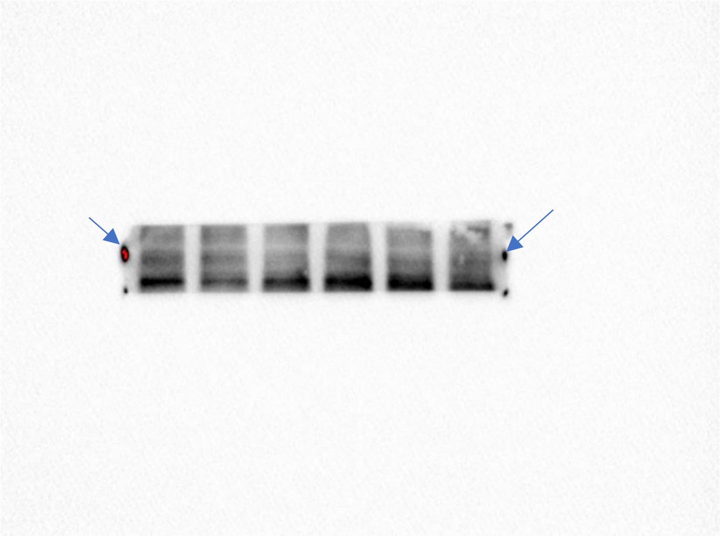

Its original internal reference GPADH (blue arrow points out the standard markers)
The upper marker is 43kda, the other one is 33kda.

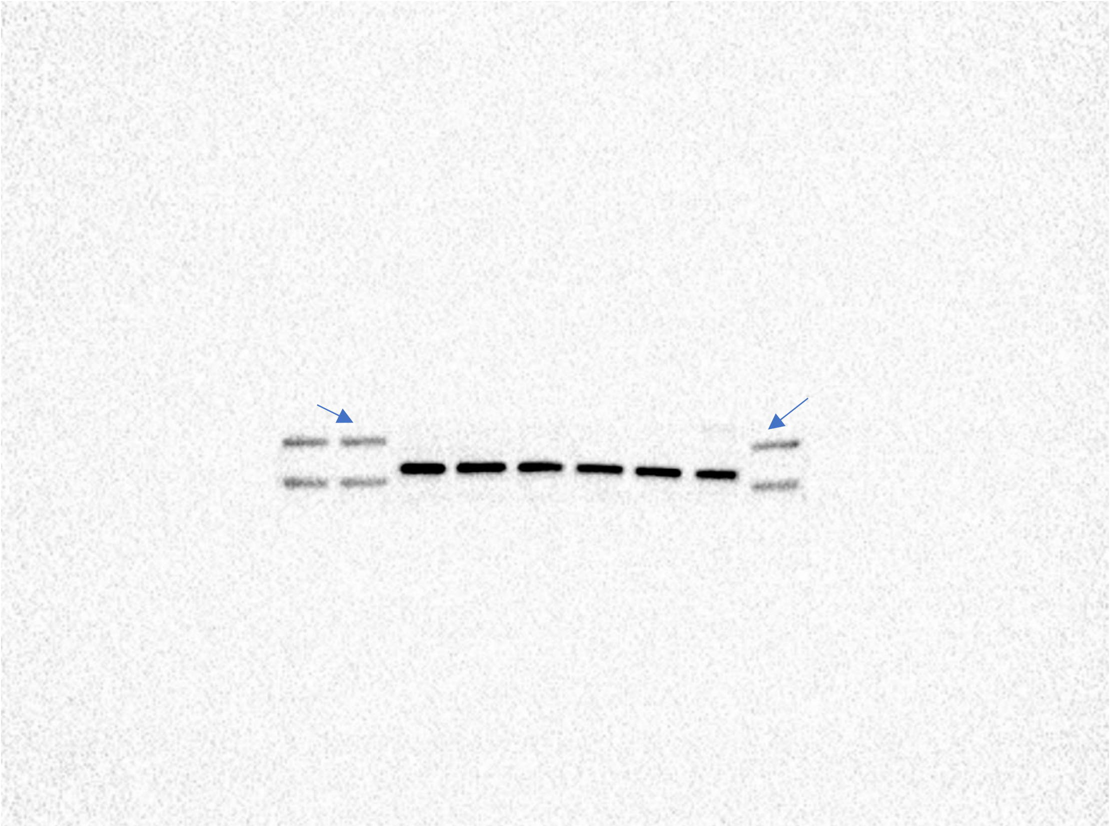

T22 Show WB picture (blue arrow points out the standard markers)

The upper marker is 55kda, the other one is 43kda.

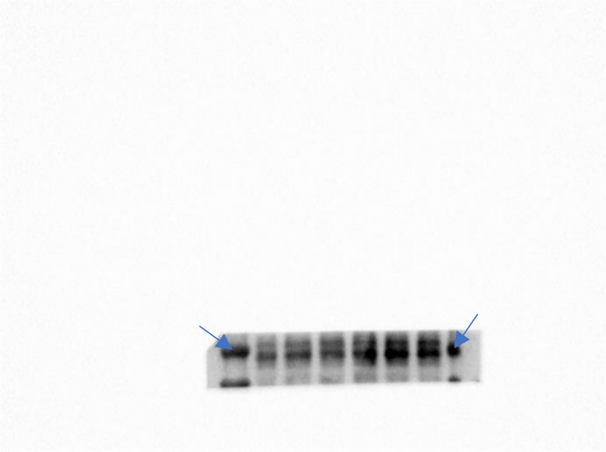

Its original internal reference GPADH (blue arrow points out the standard markers)

The upper marker is 43kda, the other one is 33kda.

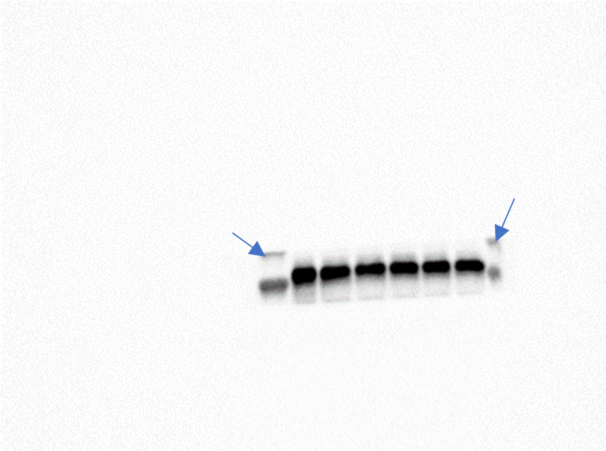

T22 WB picture for statistical analysis (blue arrow points out the standard markers)

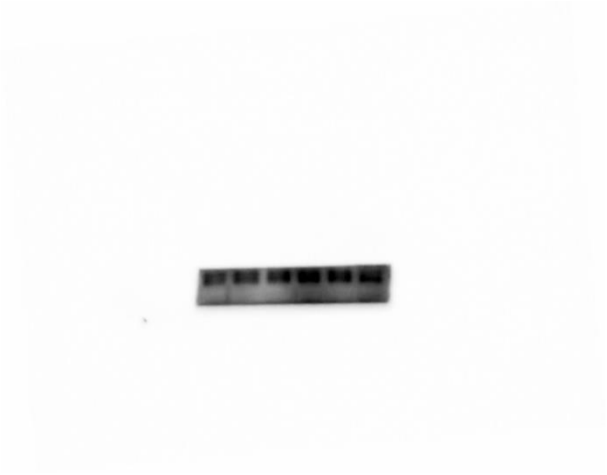

Its original internal reference GPADH (blue arrow points out the standard markers)
The marker is 33kda.

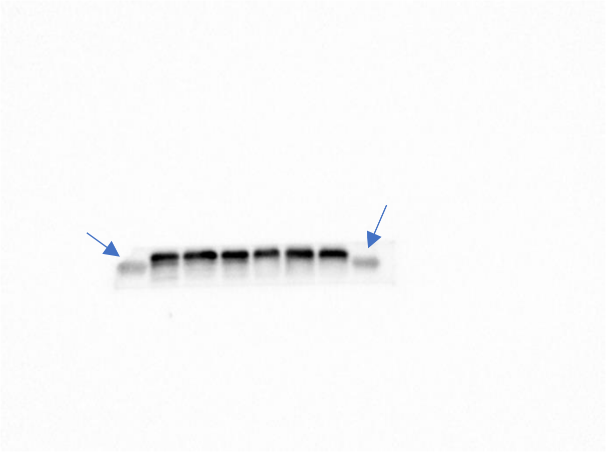

LM G3BP1 Show WB picture (blue arrow points out the standard markers)
The upper marker is 72kda, the other one is 55kda.

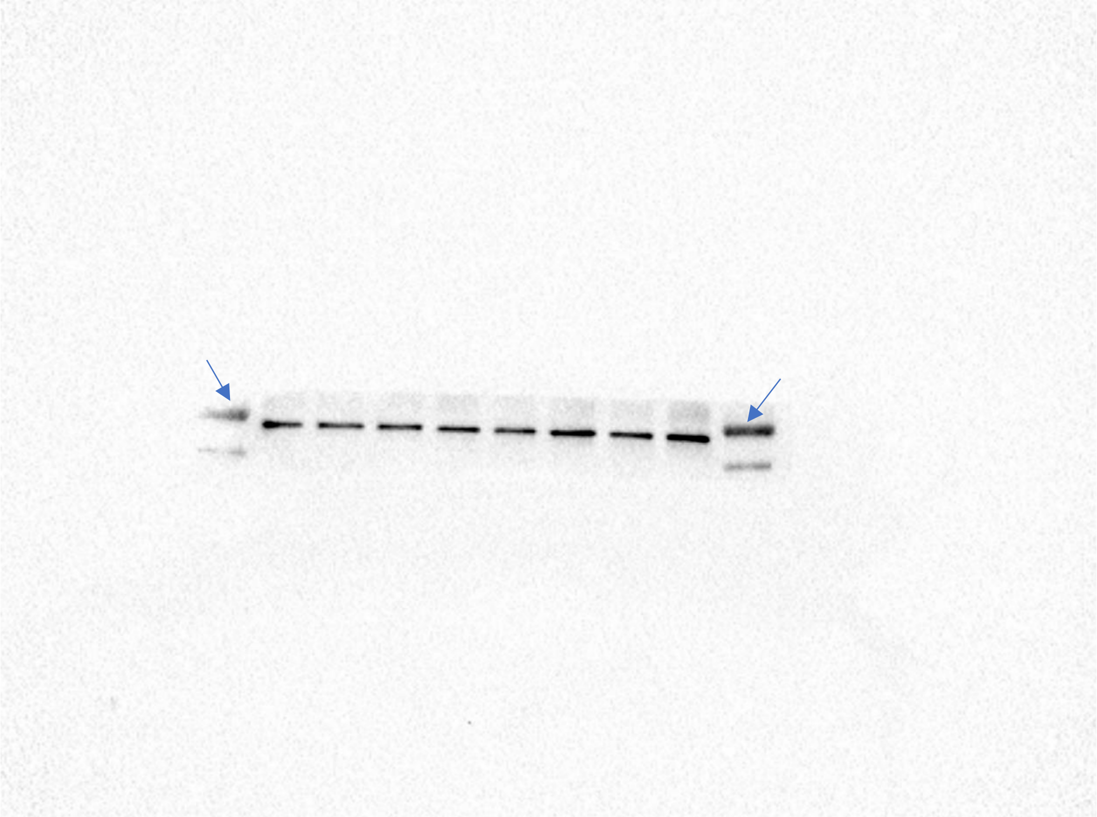

Its original internal reference GPADH (blue arrow points out the standard markers)
The upper marker is 43kda, the other one is 33kda.

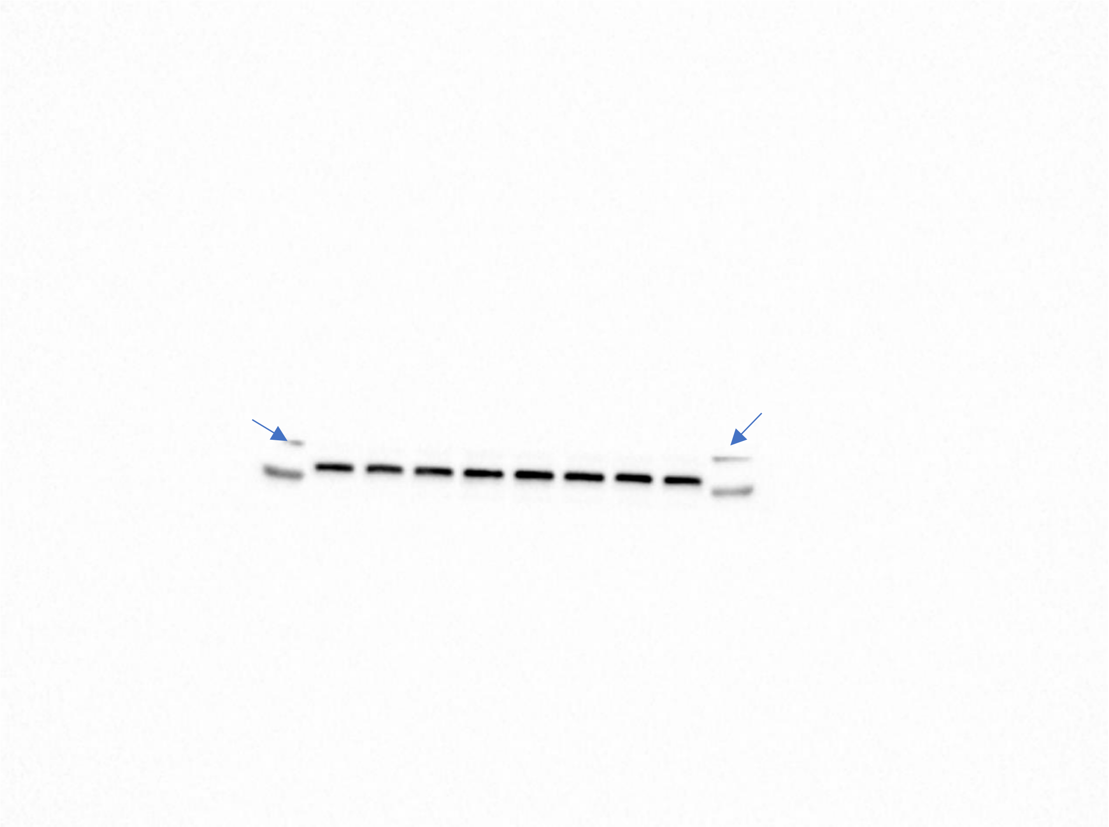

LM G3BP1 WB picture for statistical analysis (blue arrow points out the standard markers)
The upper marker is 72kda, the other one is 55kda.

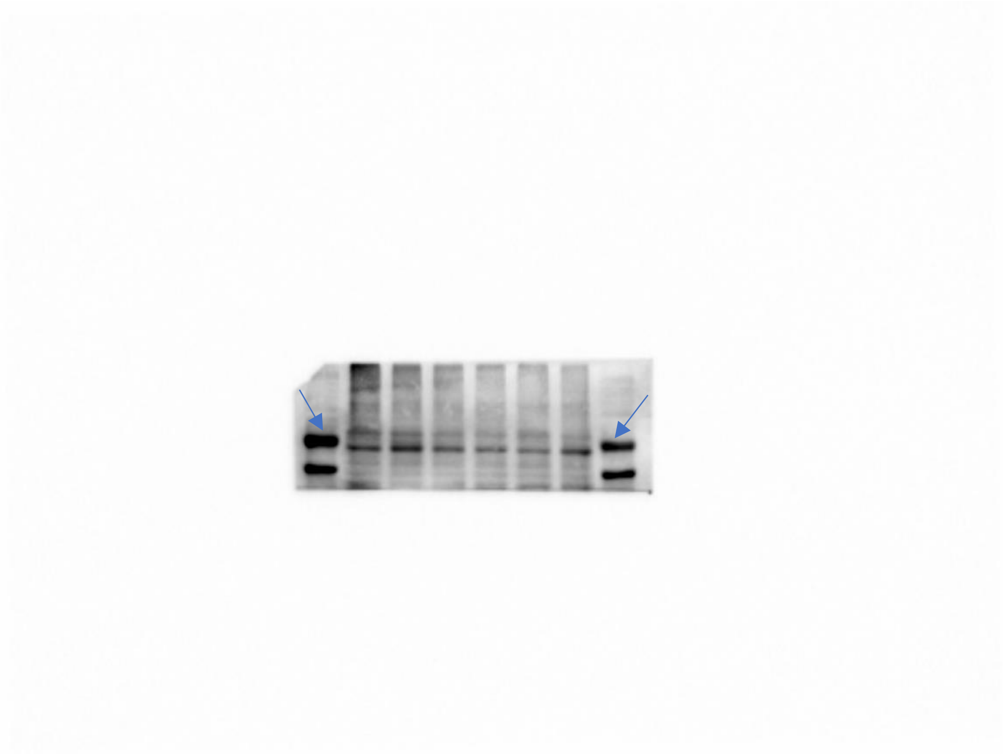

Its original internal reference GPADH (blue arrow points out the standard markers)
The upper marker is 43kda, the other one is 33kda.

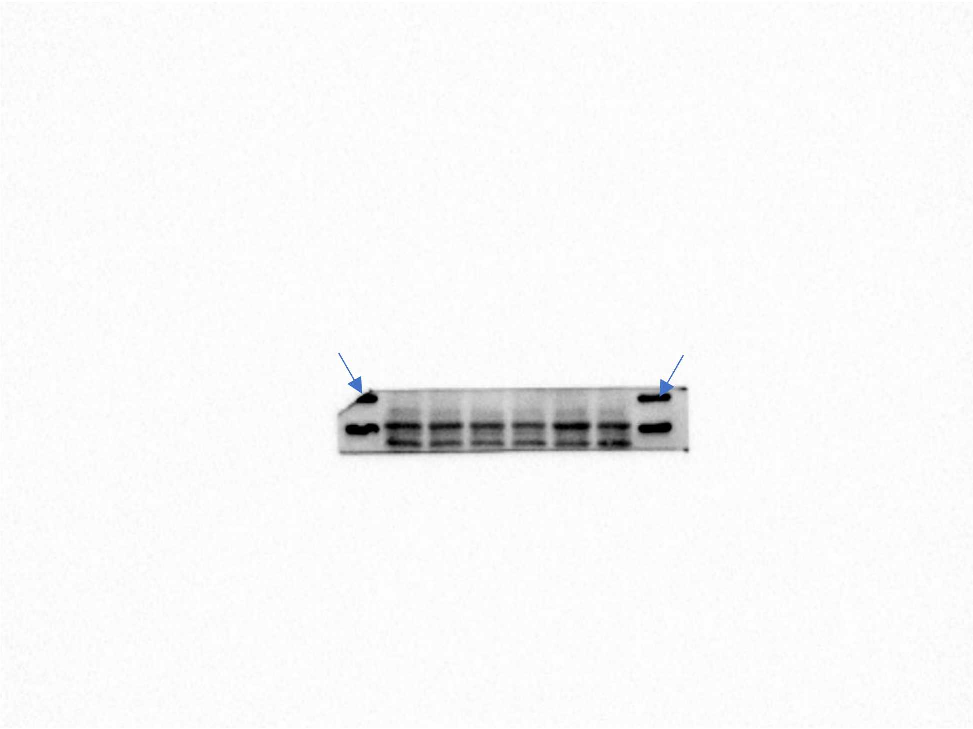

AD G3BP1 Show WB picture (blue arrow points out the standard markers)

The marker is 72kda

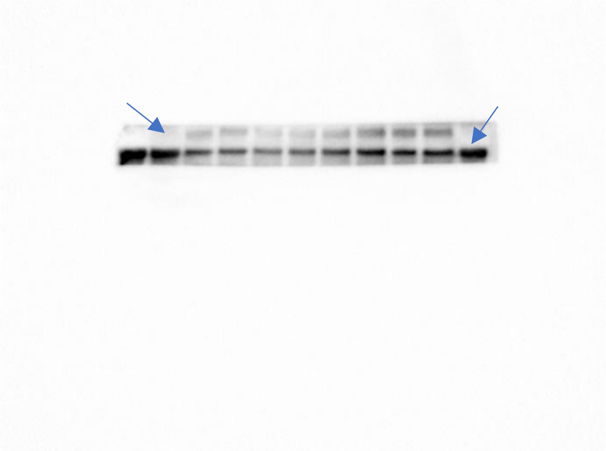

Its original internal reference GPADH (blue arrow points out the standard markers)

The upper marker is 43kda, the other one is 33kda.

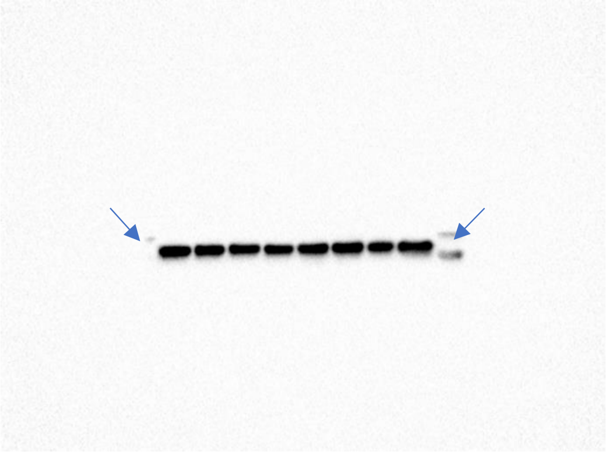

AD G3BP1 WB picture for statistical analysis (blue arrow points out the standard markers)
The marker is 72kda

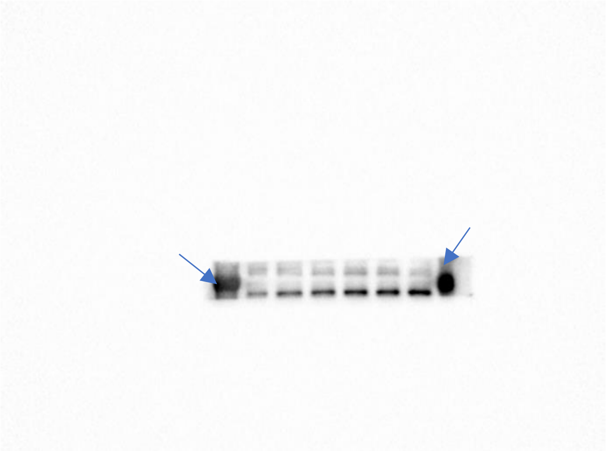

Its original internal reference GPADH (blue arrow points out the standard markers)
The upper marker is 43kda, the other one is 33kda.

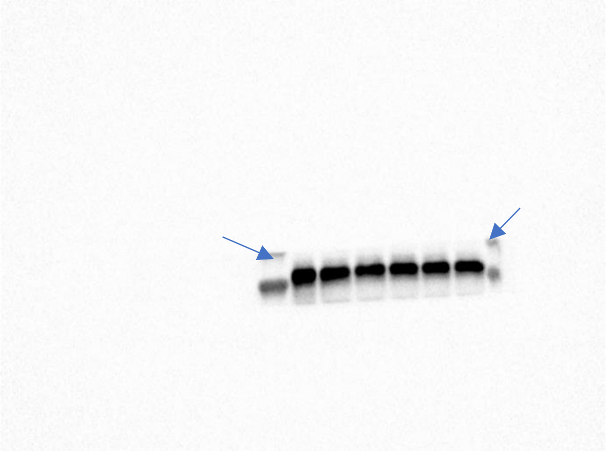

LM SYK Show WB picture (blue arrow points out the standard markers)
The marker is 72kda.

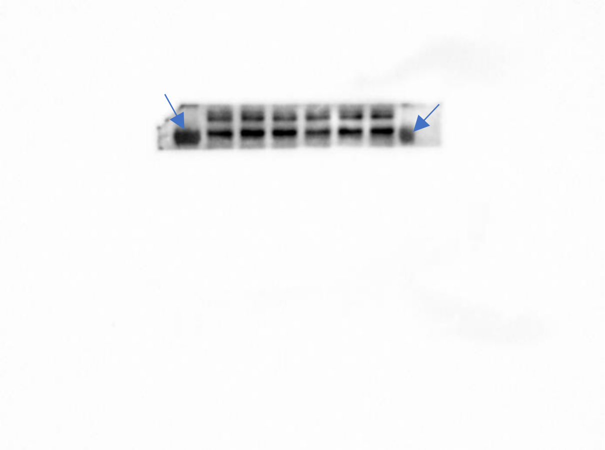

Its original internal reference GPADH (blue arrow points out the standard markers)

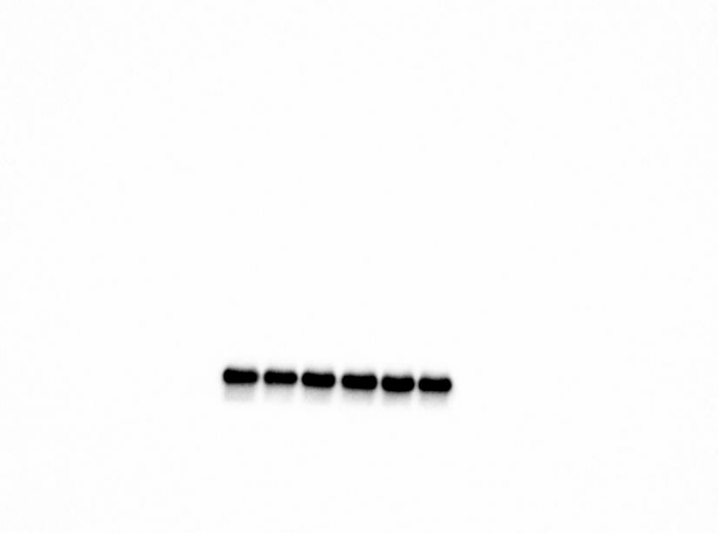

LM SYK WB picture for statistical analysis (blue arrow points out the standard markers)
The marker is 72kda.

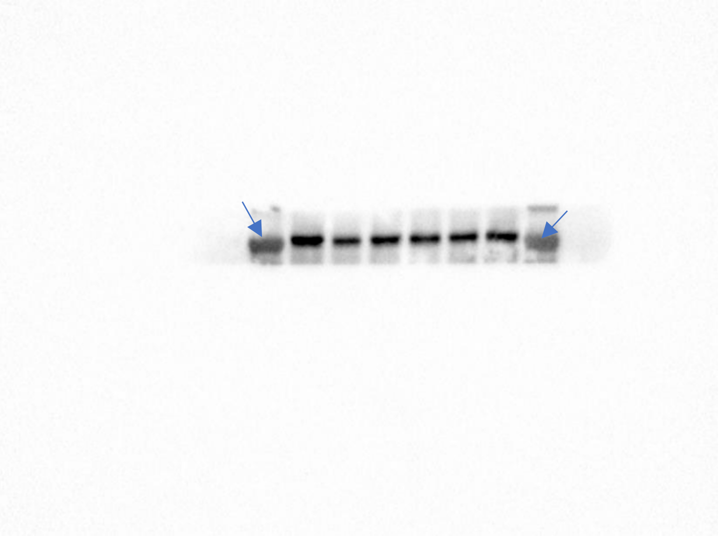

Its original internal reference GPADH (blue arrow points out the standard markers)

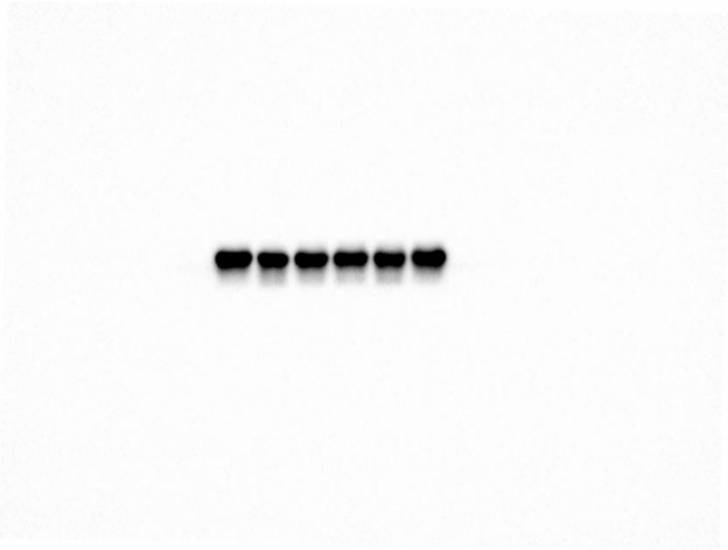

AD SYK Show WB picture (blue arrow points out the standard markers)

The marker is 72kda.

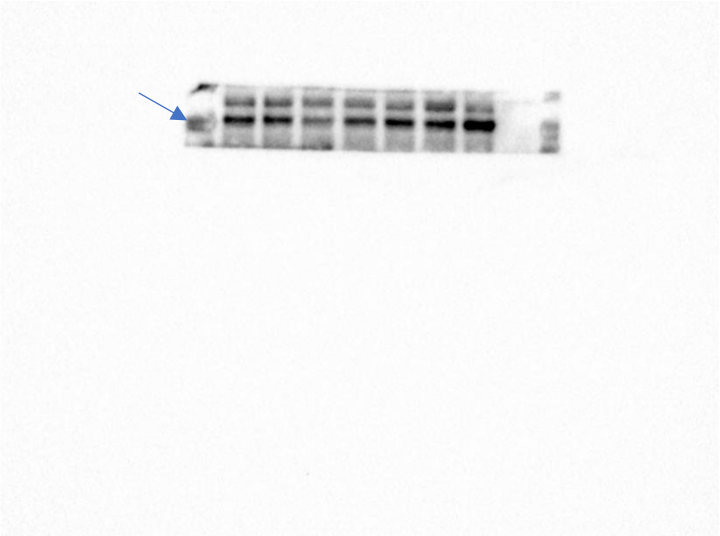

Its original internal reference GPADH (blue arrow points out the standard markers)

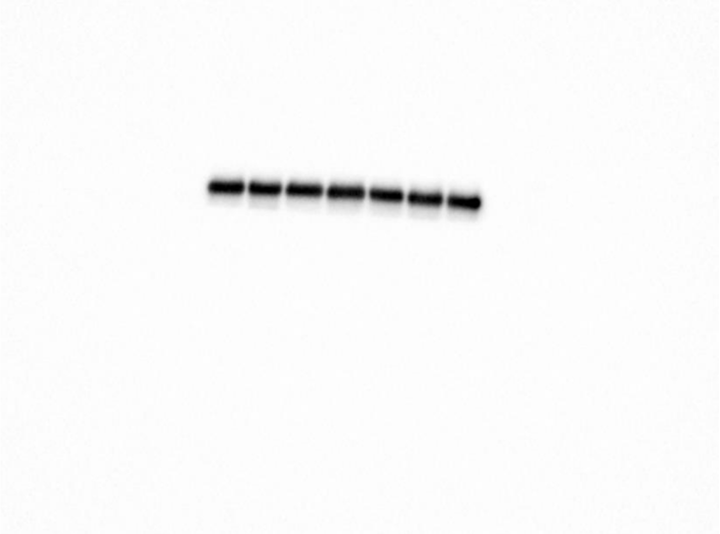

AD SYK WB picture for statistical analysis (blue arrow points out the standard markers)
The marker is 72kda.

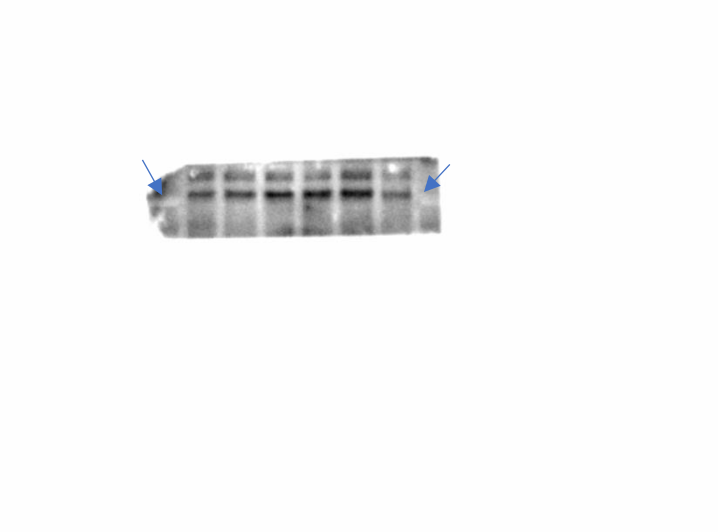

Its original internal reference GPADH (blue arrow points out the standard markers)

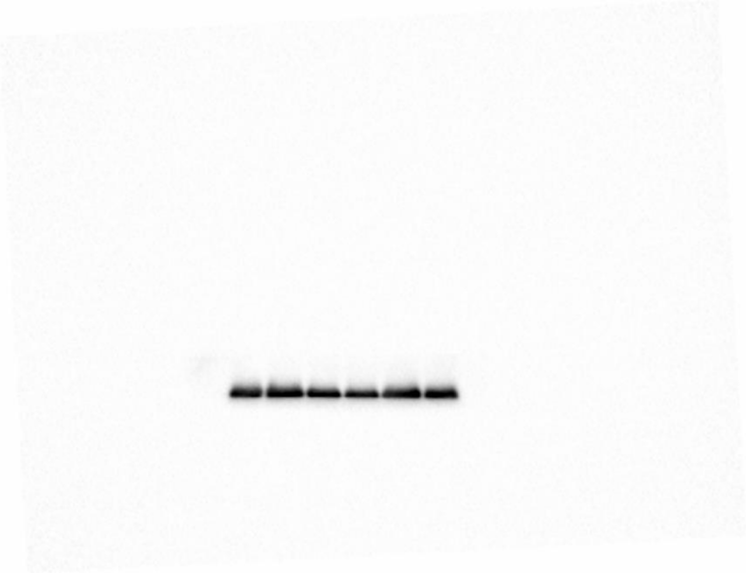

LM p-SYK Show WB picture (blue arrow points out the standard markers)
The marker is 72kda.

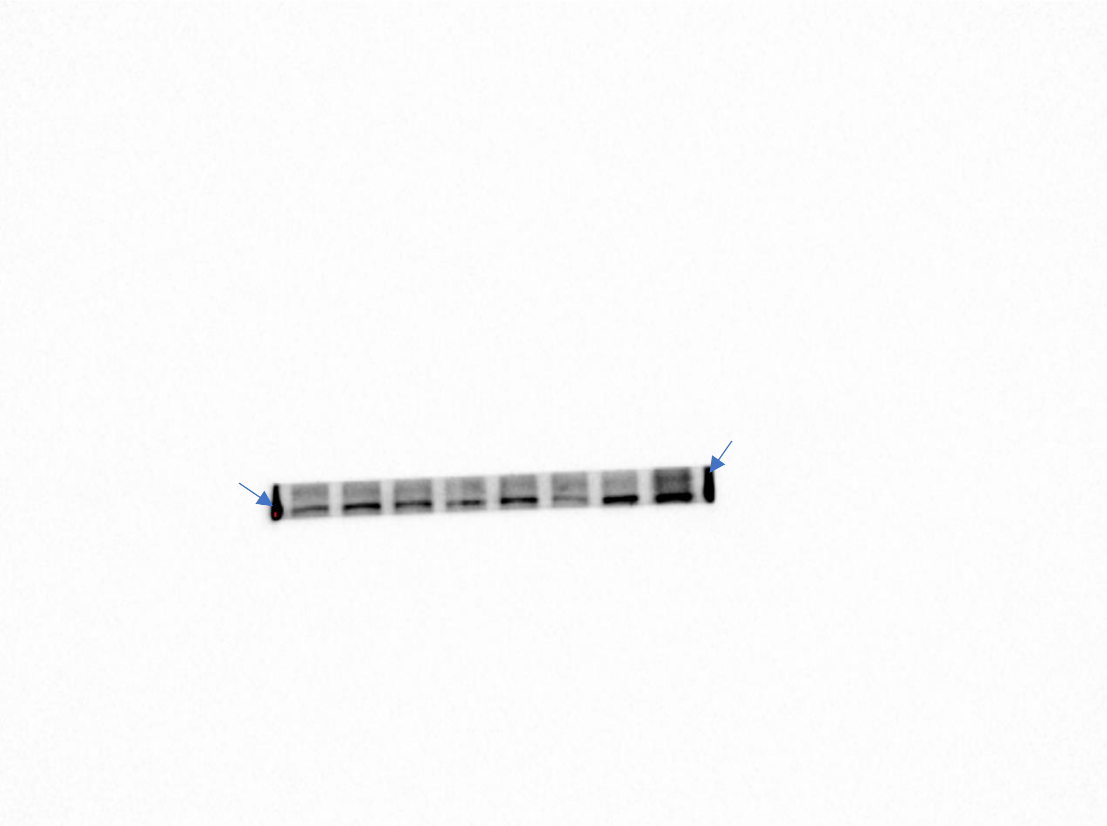

Its original internal reference GPADH (blue arrow points out the standard markers)
The upper marker is 43kda, the other one is 33kda.

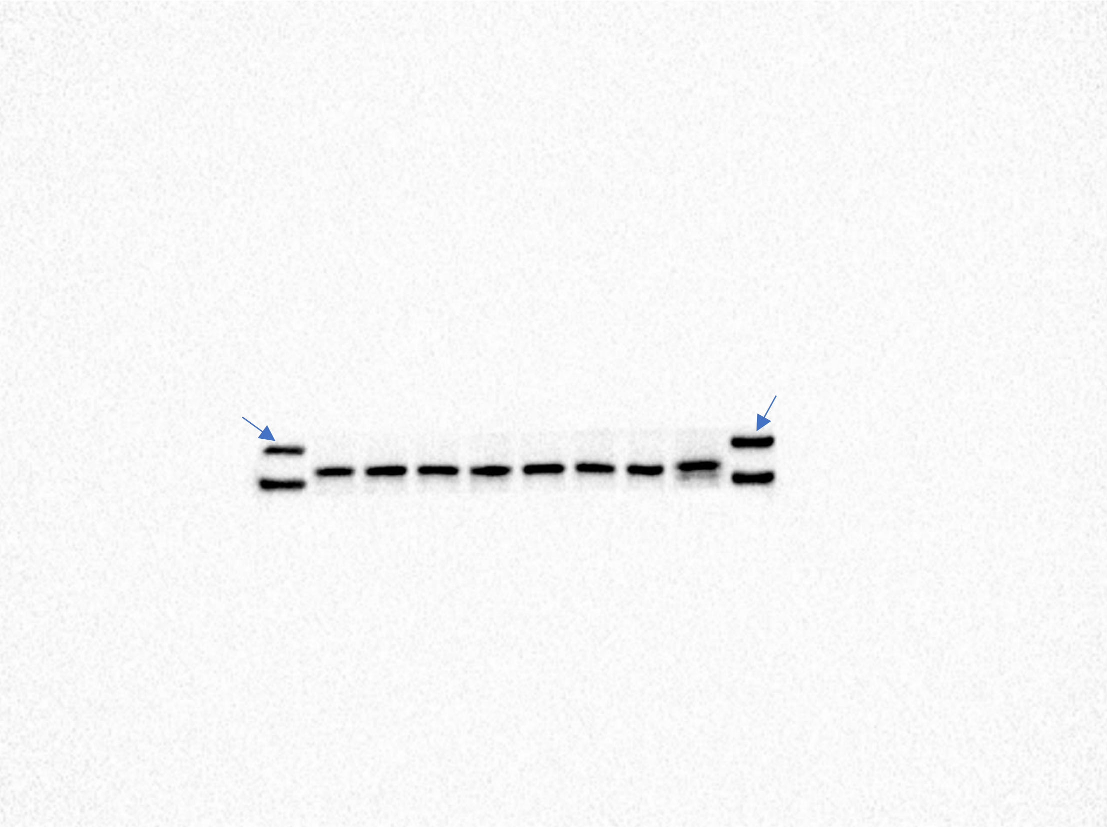

LM p-SYK WB picture for statistical analysis (blue arrow points out the standard markers)
The upper marker is 72kda, the other one is 55kda.

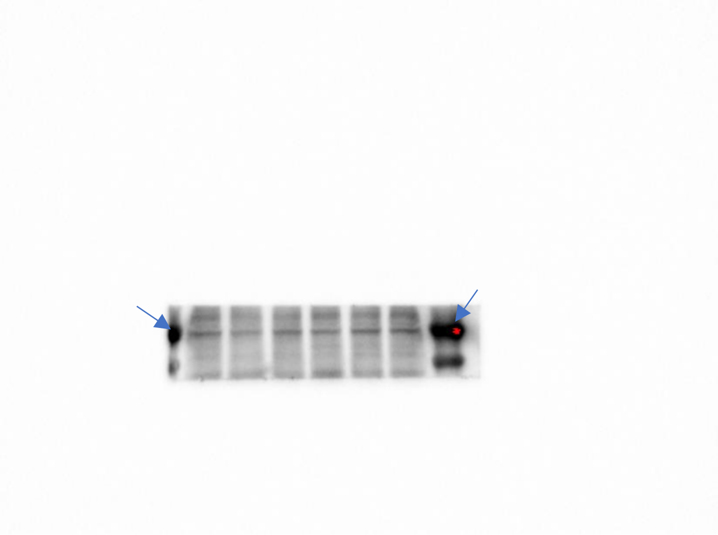

Its original internal reference GPADH (blue arrow points out the standard markers)
The upper marker is 43kda, the other one is 33kda.

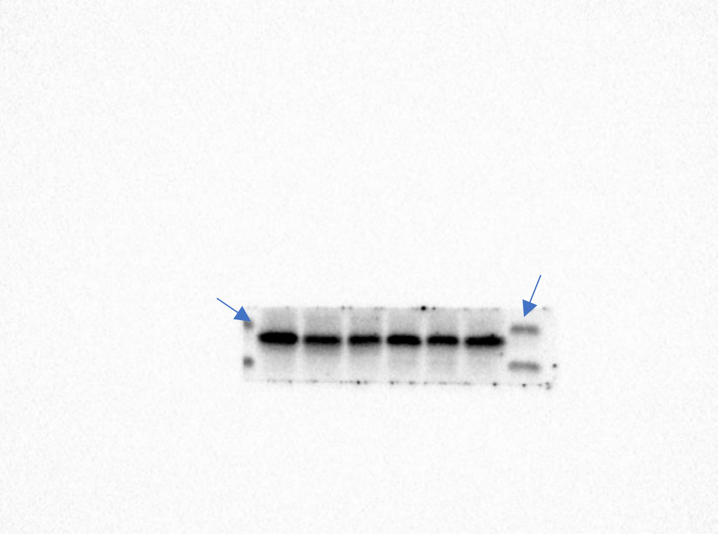

AD p-SYK Show WB picture(blue arrow points out the standard markers)

The marker is 72kda.

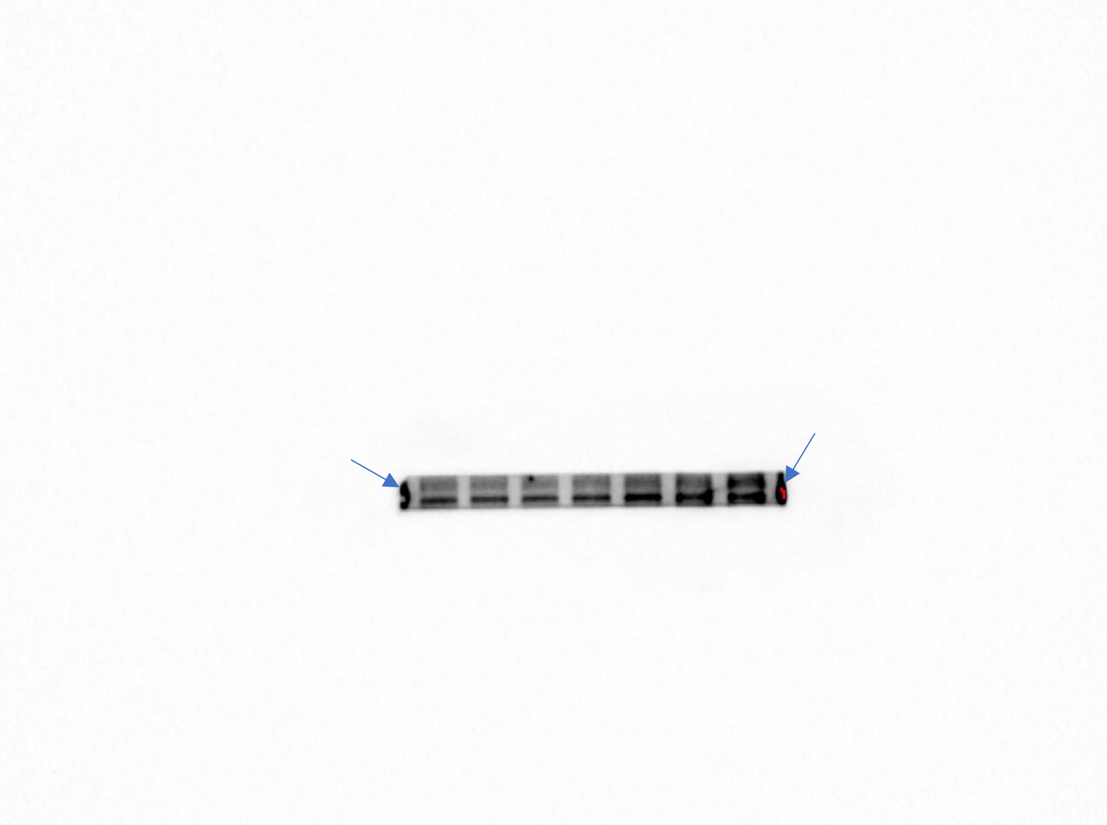

Its original internal reference GPADH (blue arrow points out the standard markers)

The marker is 43kda and 33kda.

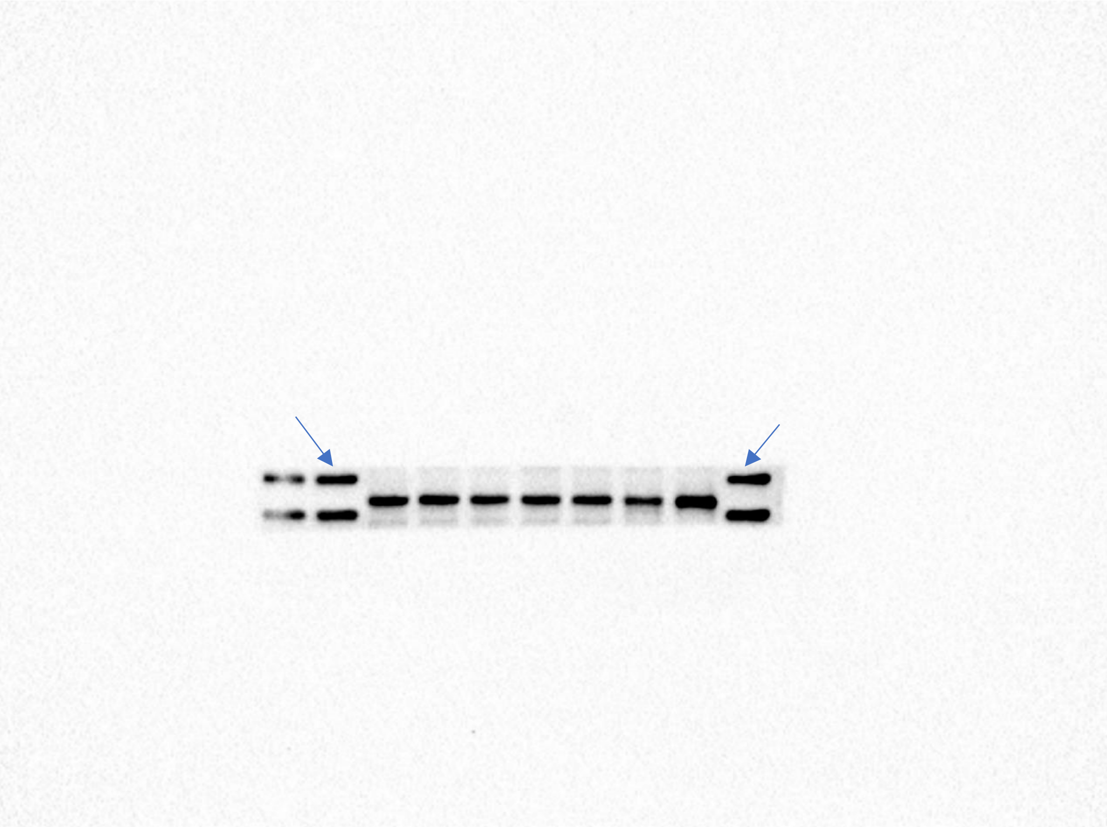

AD p-SYK WB picture for statistical analysis (blue arrow points out the standard markers)

The marker is 72kda.

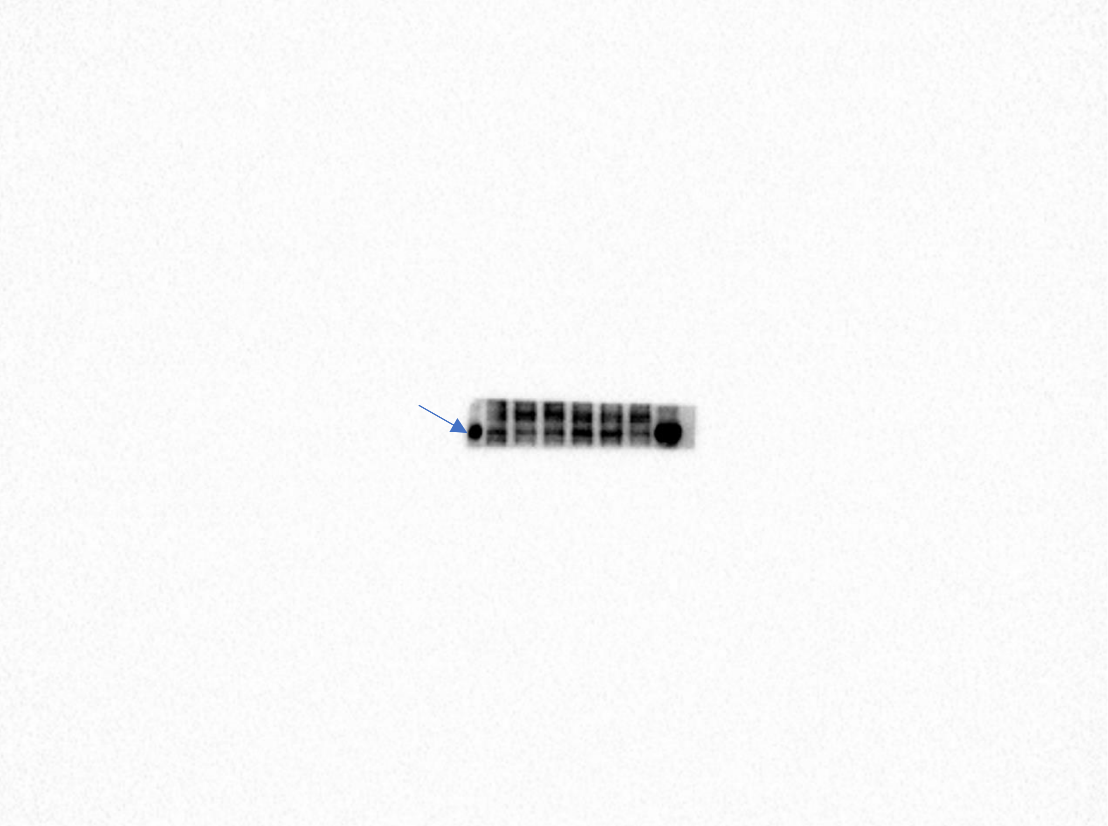

Its original internal reference GPADH (blue arrow points out the standard markers)

The marker is 33kda.

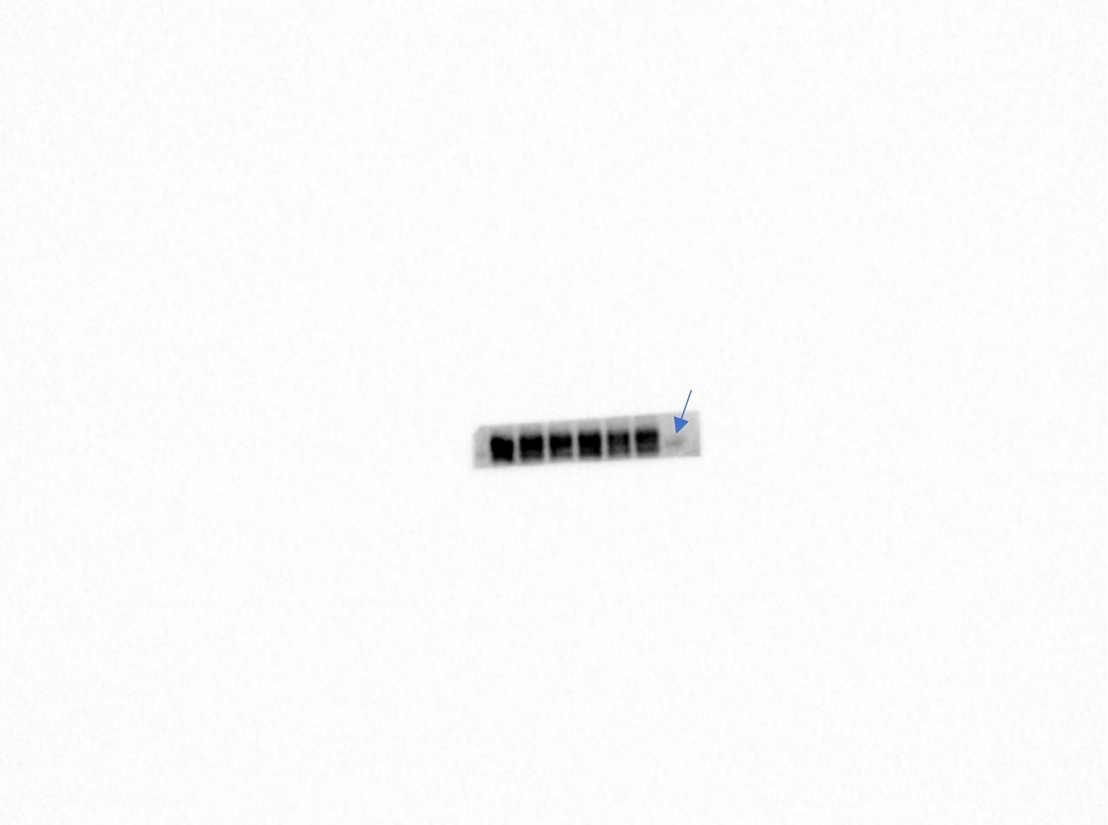

Supplement: Supplementary file 1 [file Data_Sheet_1.PDF]
